# Supplementary material for: A magma ocean origin to divergent redox evolutions of rocky planetary bodies and early atmospheres
Source: Nat Commun. 2020 Apr 24;11:2007. doi: 10.1038/s41467-020-15757-0 (PMC7181735; doi:10.1038/s41467-020-15757-0)
Supplement: Supplementary file 1 — Supplementary Information [file 41467_2020_15757_MOESM1_ESM.pdf]

Supplementary Information for

A magma ocean origin to divergent redox evolutions of rocky planetary bodies and early atmospheres

By

Deng et al.

Supplementary Figures

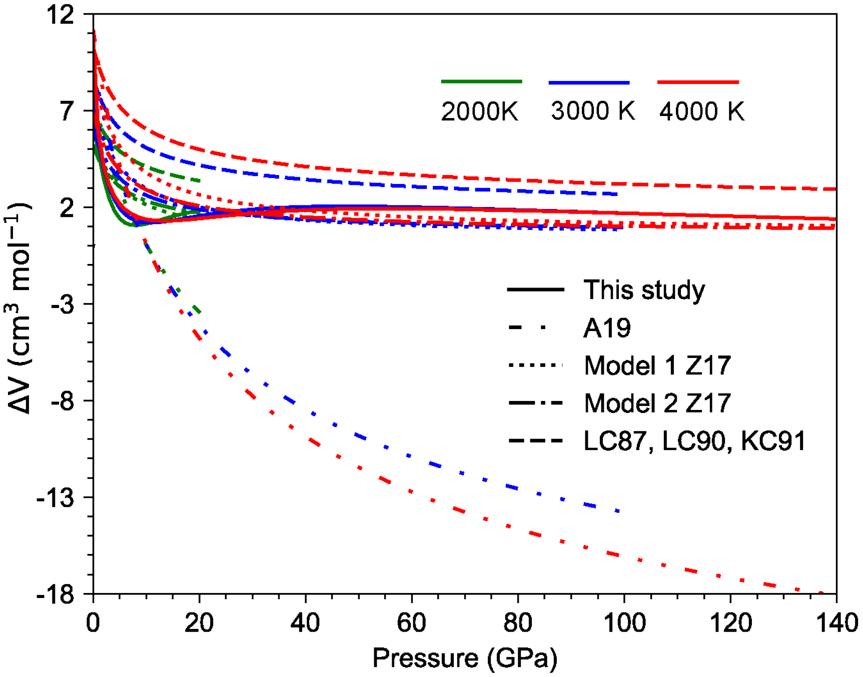

Supplementary Figure 1. Molar volume difference of $FeO_{1.5}$ and FeO, ΔV of silicate melts containing 12.5 mol% iron at 2000, 3000, and 4000 K. A19, Z17, LC87, LC90, KC91 denote Armstrong et al.[4], Zhang et al. [5], Lange and Carmichael [6,7], and Kress and Carmichael [8], respectively. Volumes are plotted along isotherms only to pressures where the simulated systems were in a molten state as confirmed by examination of radidal distribution functions and mean square displacement plots.

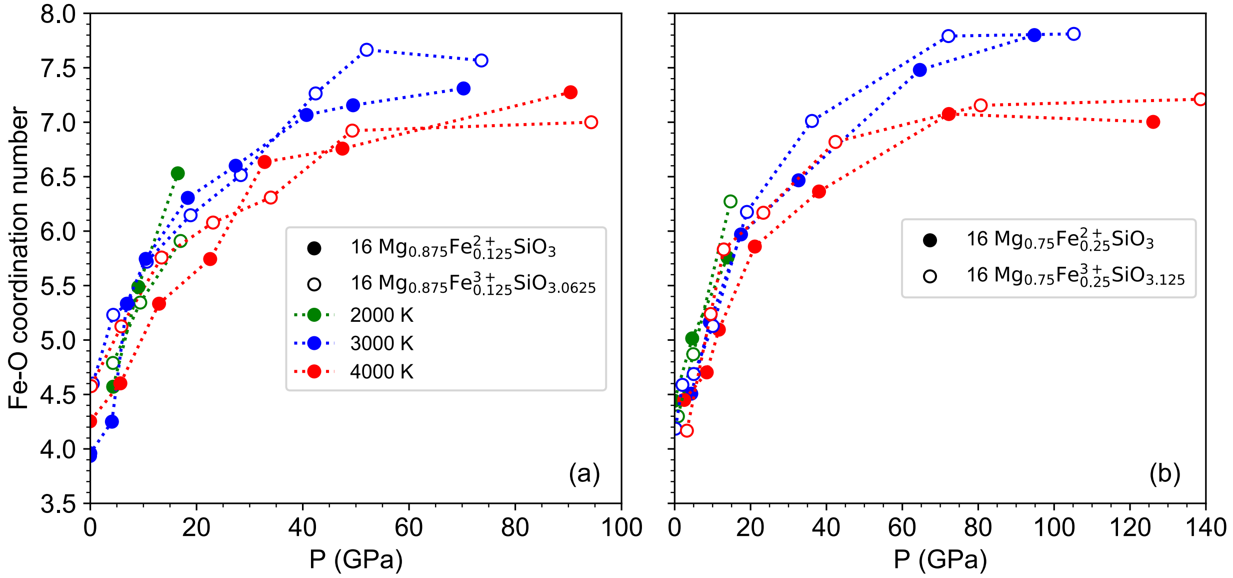

Supplementary Figure 2. Average Fe-O coordination number of silicate melt at 2000 K (green), 3000 K (blue) and 4000 K (red). (a) silicate melt with 12.5 mol% iron. **(b)** 25 mol% iron. Melts containing Fe^{2+} and Fe^{3+} are represented using solid and open symbols respectively. The mean Fe-O coordination numbers ($CN_{\text{Fe-O}}$) are calculated for all silicate melts simulated. At 0 GPa, $CN_{\text{Fe-O}}$ lies between 4 and 4.5 for both Fe^{2+} and Fe^{3+}, in agreement with the experimental results on ferrosilicate glasses and melts [9-12]. Compression densifies the melts and increases the coordination. $CN_{\text{Fe-O}}$ of both Fe^{2+} and Fe^{3+} rapidly increases initially up to 20 GPa and gradually levels out after ~40 GPa. These pressure ranges correspond well to the sharp decrease of the volume difference of $FeO_{1.5}$ and FeO and its plateau, thus indicating a good correlation between the local coordination environment of Fe and its partial molar volume. The predicted sharp increasing trend of $CN_{\text{Fe-O}}$ at low pressures is also in qualitative good agreement with the experimental measurements on fayalite melt[13]. Zhang et al.[5] studied andesitic glasses observed a similar increasing trend with pressure for the coordination of Fe^{2+}-O but no noticeable change of Fe^{3+}-O at pressures lower than ~7 GPa. This apparent discrepancy for Fe^{3+}-O may be due to the intrinsic difference of glasses and melts or the bulk composition difference between our simulation and previous experiments[5].

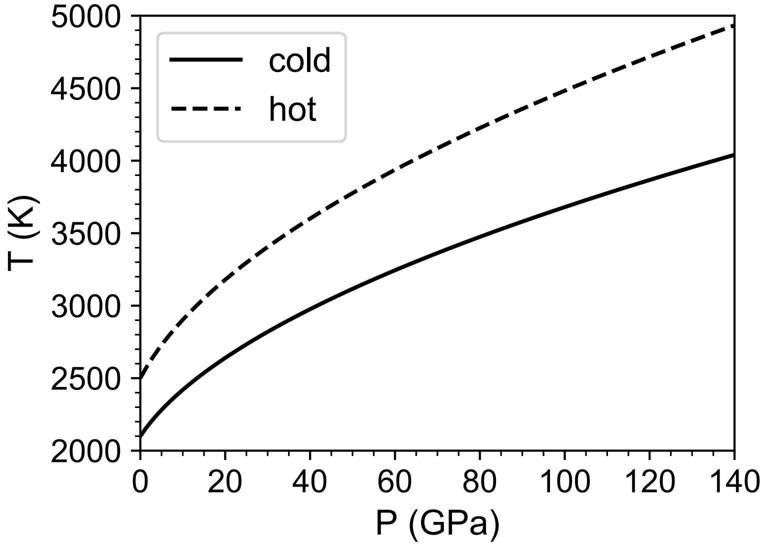

Supplementary Figure 3. Adiabatic temperature profiles of the magma ocean, assuming surface temperature of 2100 K (sold curve) and 2500 K (dashed curve). These hot and cold temperature profiles represent two end-member cases for a magma ocean. The adiabatic temperature profile of the MOs is related to the Grüneisen parameter (ξ) by $\xi = (\partial \ln T / \partial \rho)_s$, where T is temperature and ρ is density. We use the equation of state of $MgSiO_3$ melt by ref.[1] and ref.[2] to describe the variation of density of the magma with temperature. We consider these two thermal profiles following ref.[3].

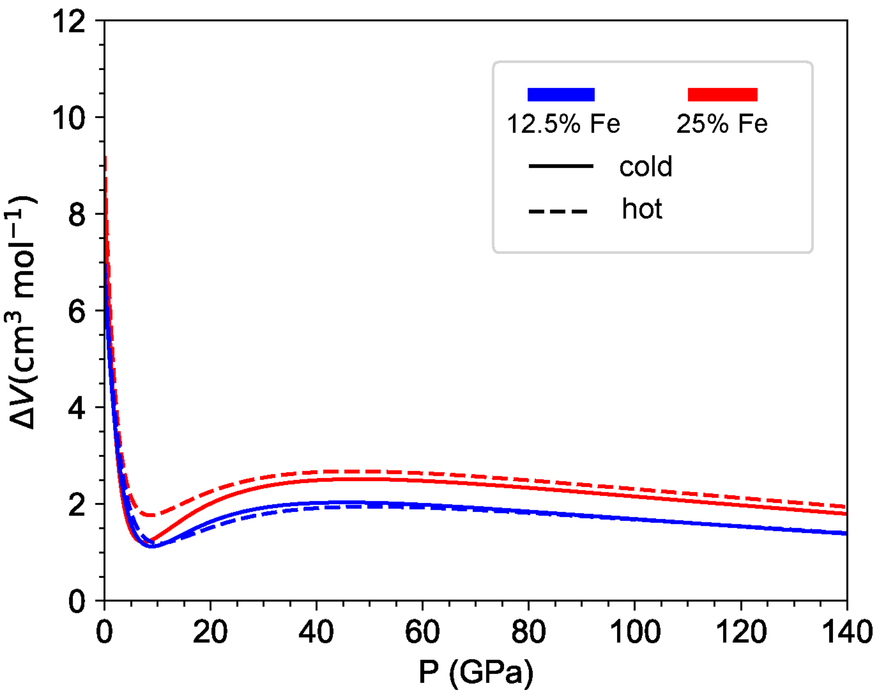

Supplementary Figure 4. Molar volume difference of $FeO_{1.5}$ and FeO, ΔV of silicate melts containing 12.5 mol% iron (blue curves) and 25 mol% iron (red curves) along the cold (solid) and hot (dashed) temperature profiles of MOs.

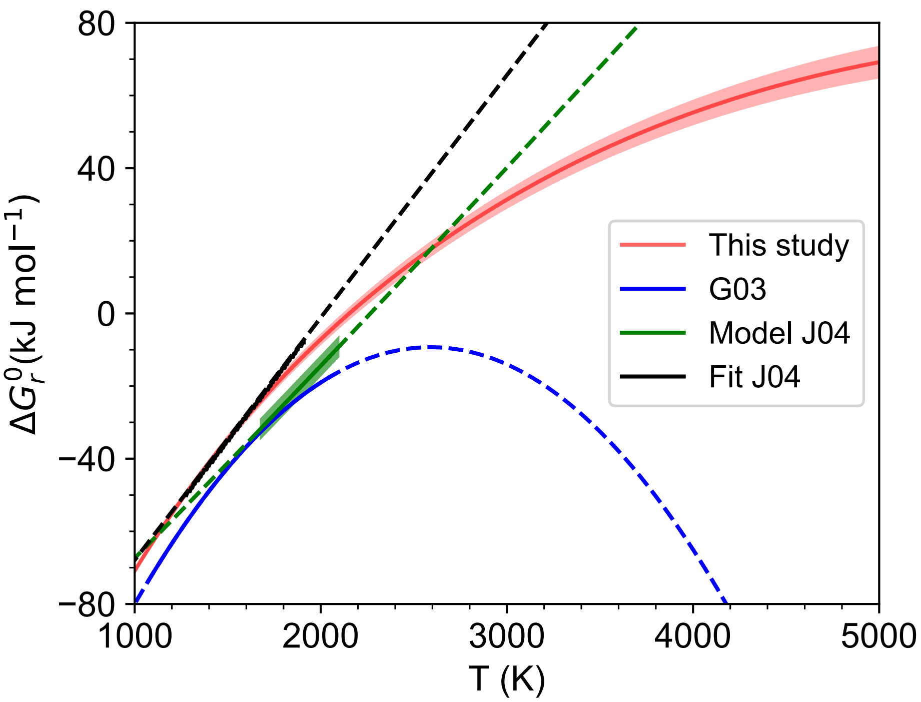

Supplementary Figure 5. Gibbs free energy of the reaction (1), $\Delta G_{\mathrm{r}}^{0}(T)$ as a function of temperature at 1 bar. The shaded regions reflect standard uncertainties where given. Previous results of $\Delta G_{\mathrm{r}}^{0}(T)$ are also shown for comparison with dashed curves being the extrapolation outside of data and model parameters[14,15]. The uncertainties of the thermodynamic model by ref.[15] and fitted results by ref.[14] were not given in the source. G03 and J04 denote ref.[15] and ref.[14], respectively.

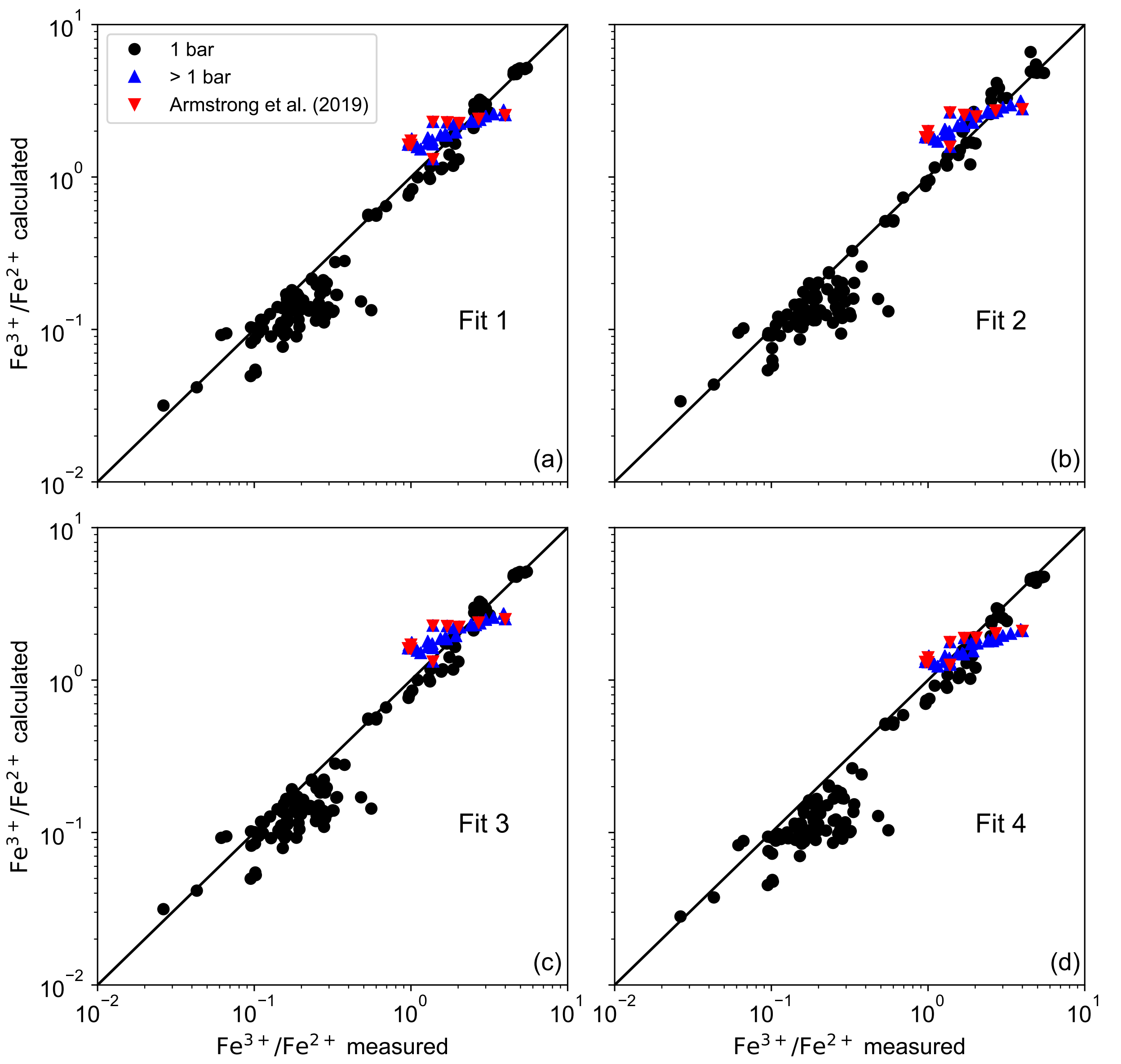

Supplementary Figure 6. Fe^{3+}/Fe^{2+} measured with those calculated using Eq. (3) with best-fitting interaction parameters (Supplementary Table 5). **(a)** and **(c)** are Fit 1 and Fit 3 excluding the 8 high-pressure data by ref.[4]. **(b)** and **(d)** are Fit 2 and Fit 4 including the 8 high-pressure data by ref. [4]. The interactions parameters, $W_{\mathrm{FeO_{1.5}-NaO_{0.5}}} - W_{\mathrm{FeO-NaO_{0.5}}}$, $W_{\mathrm{FeO_{1.5}-PO_{2.5}}} - W_{\mathrm{FeO-PO_{2.5}}}$, and $W_{\mathrm{FeO_{1.5}-TiO_2}} - W_{\mathrm{FeO-TiO_2}}$ are set to 0 in (c) and (d). High pressure data (> 1bar) by ref. [5,16] and ref. [4] are shown in blue and red triangles respectively.

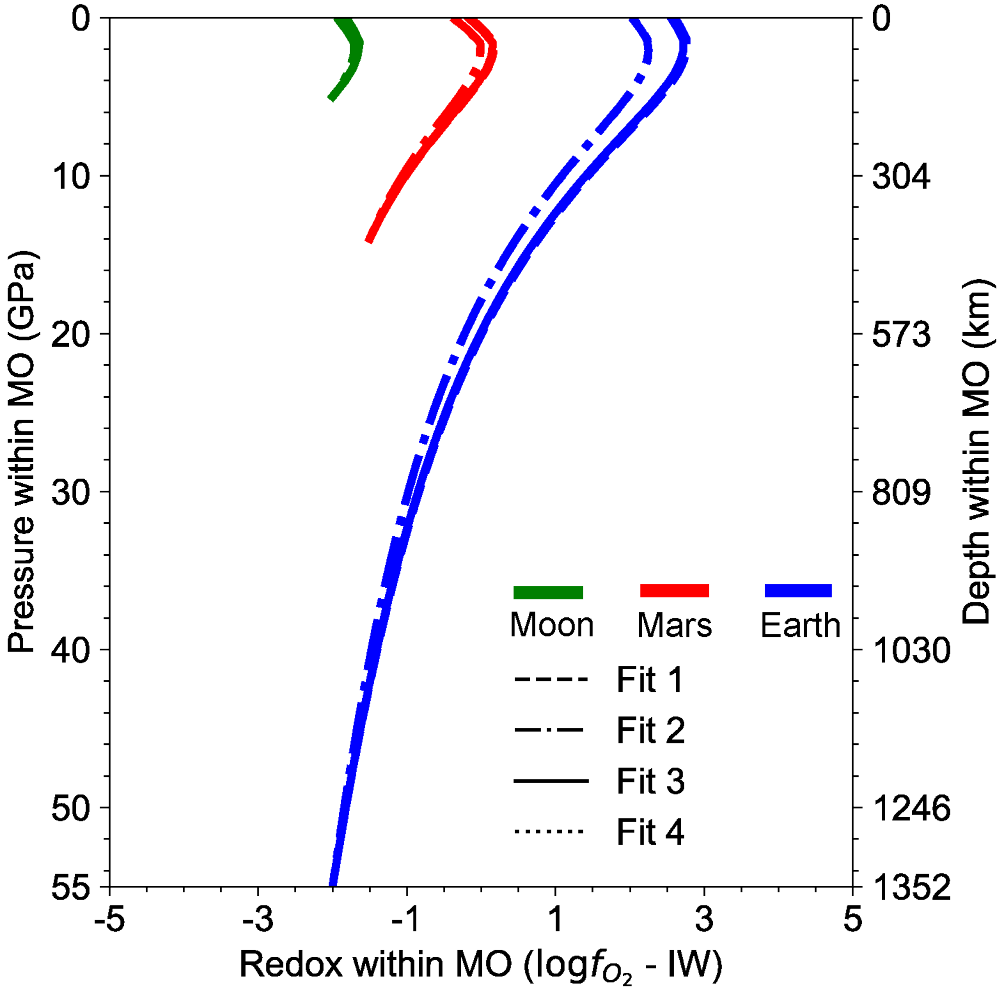

Supplementary Figure 7. Effects of fitting methods on the redox profiles of magma oceans (MOs) for Earth (blue), Mars (red), and the Moon (green) along the cold magma ocean temperature profile. The oxygen fugacity results with different sets of interaction parameters resolved by Fit 1 (dashed), Fit 2 (dashed-dotted), Fit 3 (solid), and Fit 4 (dotted) almost overlap with each other.

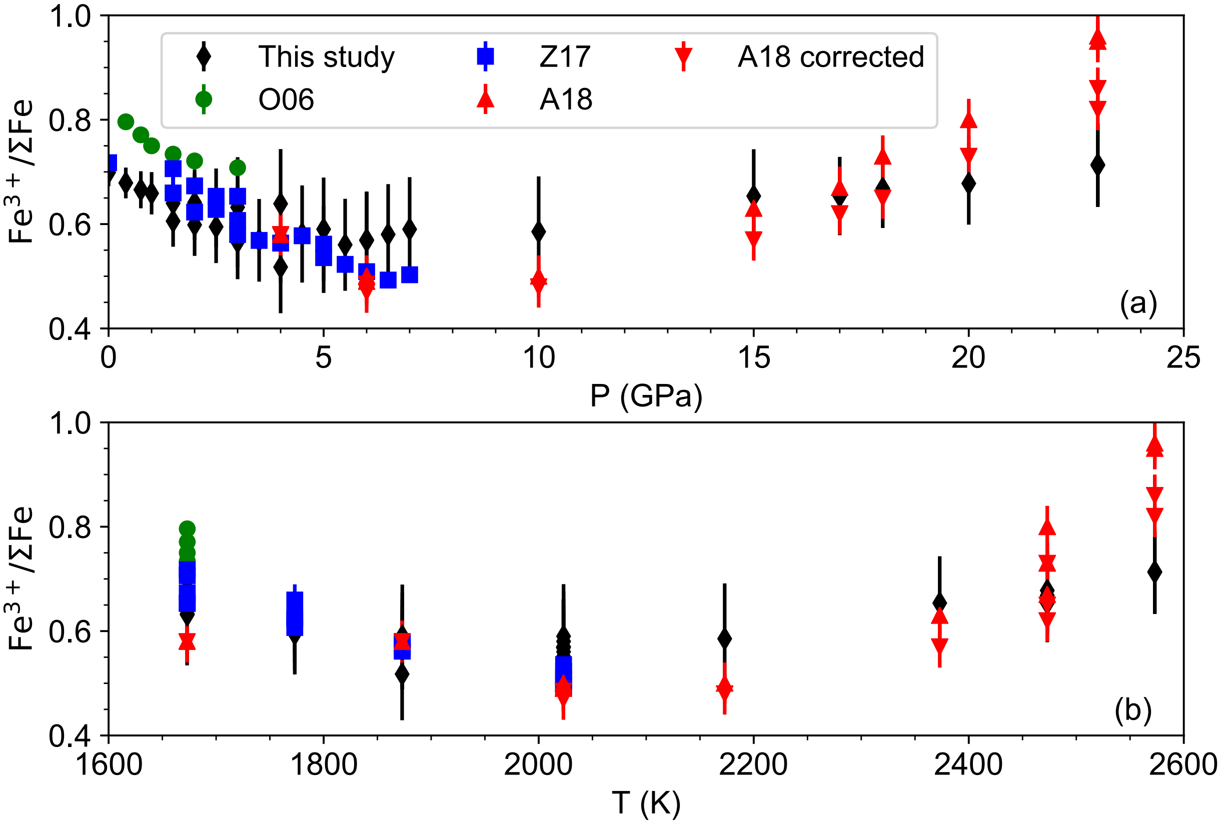

Supplementary Figure 8. Fe^{3+}/ ΣFe predicted by the best-fitting model (Fit 3, black symbols) and high temperature experimental data by O'Neill et al. (O16, green circles)[16], Zhang et al. (Z17 blue circles)[5], and Armstrong et al. (A19, red triangles)[4] with upward and downward triangles being the data without and with the possible Ru contribution removed.

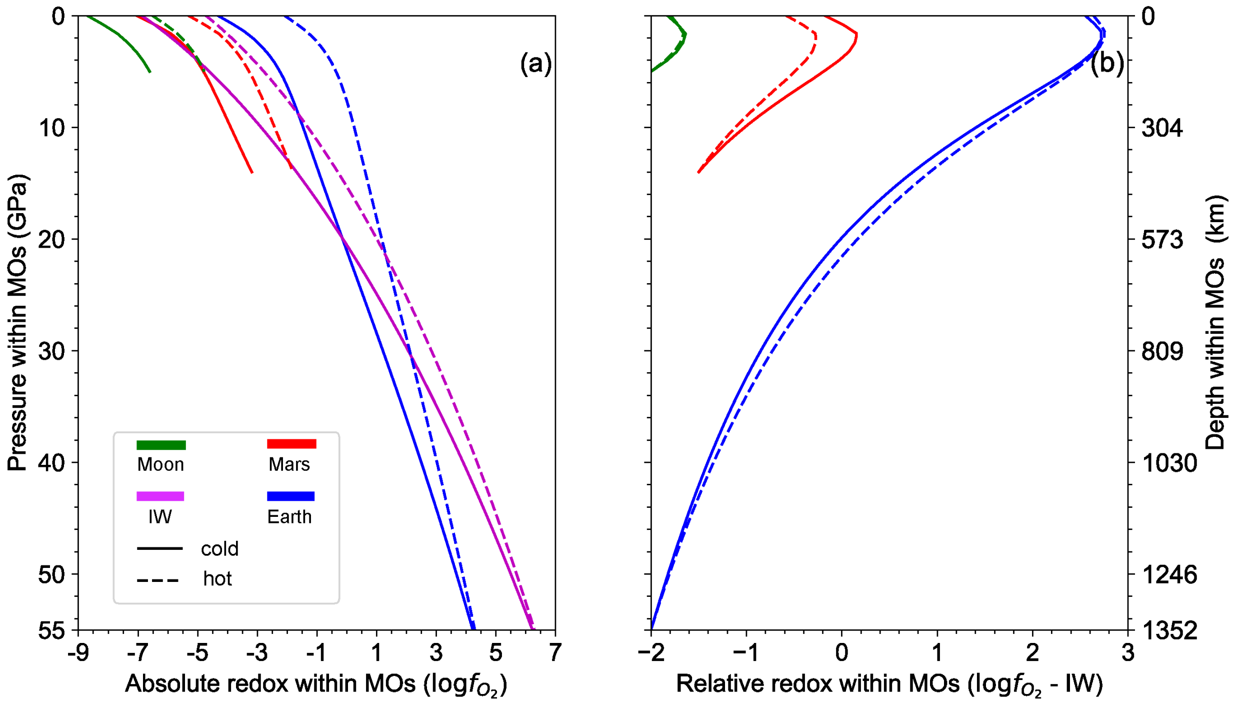

Supplementary Figure 9. Effects of temperatures on the redox profiles of magma oceans (MOs). Redox profiles of the MOs for Earth (blue), Mars (red), and the Moon (green) along both cold (solid curves) and hot thermal profiles (dashed curves). The bases of the MOs are assumed to be at 55 GPa, 14 GPa, and 5 GPa with redox states (ΔIW) of are –2, –1.5, and –2 for Earth, Mars, and the Moon, respectively. The 1σ standard deviation of the oxygen fugacity is ~0.5 log unit. **(a)** Absolute oxygen fugacities of the MOs are shown together with that of IW buffer (pink). **(b)** Oxygen fugacity relative to the IW, i.e., ΔIW = log f_{O2} – IW as a function of pressure.

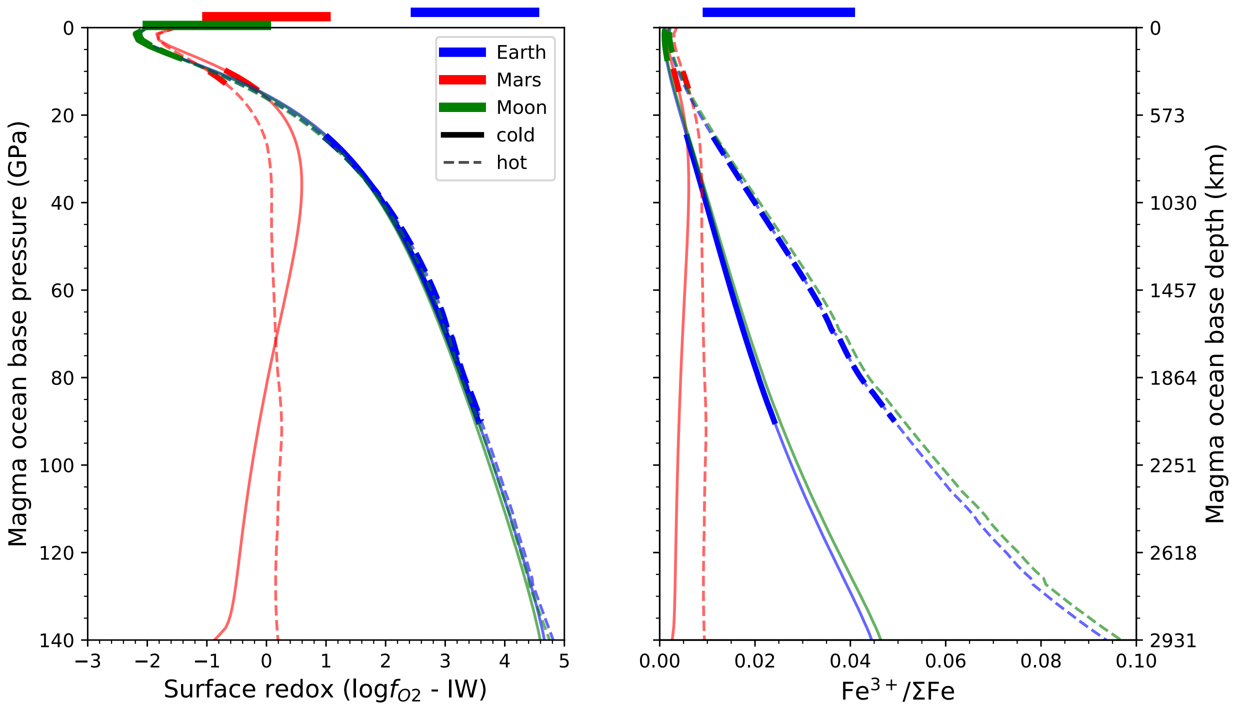

Supplementary Figure 10. Effects of magma ocean (MO) depth on the redox profiles of the surface of MOs. Relative redox state (a) and ferric iron contents (b) at the surface of MOs of Earth (blue), Mars (red), and the Moon (green) versus the pressure of the base of MOs considering both cold (solid) and hot (dashed) thermal profiles. Calculations are extended up to 140 GPa (thin curves) overlain by the plausible pressure ranges of the bases of the MOs suggested by previous studies[17-20] (thick curves). The redox state/ferric iron content of the present (upper) mantle of Earth (blue), Mars (red), and the Moon (green) are presented as horizontal bars[21,22]. The ferric iron contents of the mantle of Mars and the Moon are poorly constrained (not shown) because the available samples suffer from alterations and post-formation oxidations and cannot reflect the ferric iron contents of the source mantle[23,24].

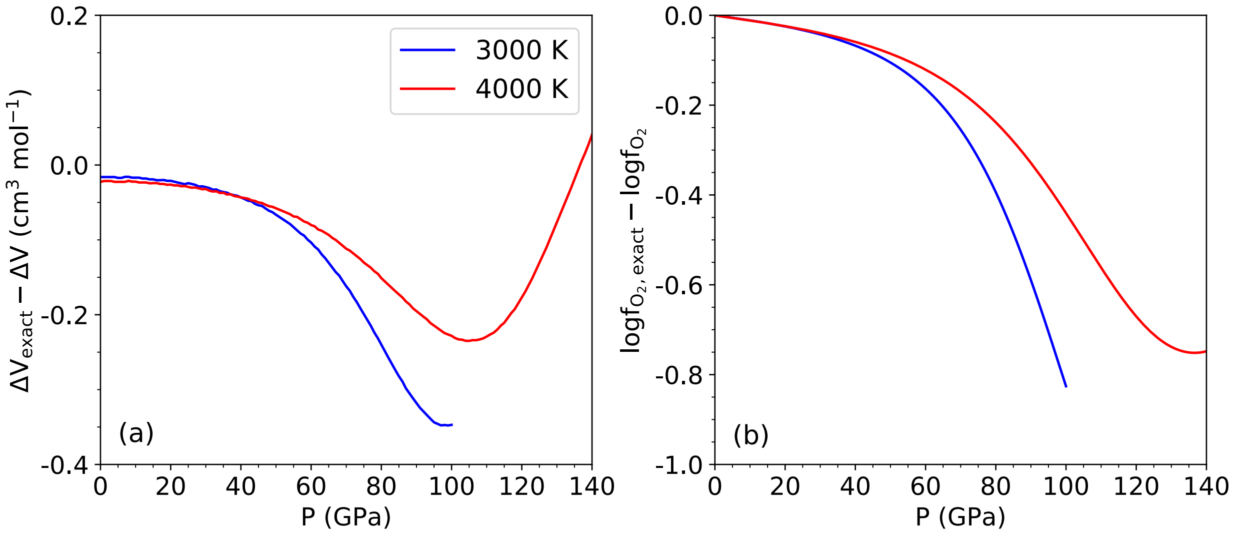

Supplementary Figure 11. Effects of spin transition of iron in silicate melts. (a) The difference between ΔV_{exact} (the molar volume difference of $FeO_{1.5}$ and FeO considering spin transition of Fe) and ΔV (the molar volume difference of $FeO_{1.5}$ and FeO considering only HS states for both Fe^{3+} and Fe^{2+}) for 25 mol% iron content **(b)** The corresponding difference in oxygen fugacity. Results are plotted along isotherms only to pressures where the simulated systems were in a liquid state.

Supplementary Notes

Supplementary Note 1

The thermodynamics of reaction (Eq. 1) has not yet been evaluated at temperatures above 2100 K for previous low temperature ΔG_r^0 models[14,15]. As the thermal profiles of the MOs are all above 2100 K (Supplementary Fig. 3), high temperature ΔG_r^0 models are required. The thermodynamic properties of liquid FeO and oxygen gas at 1 bar are readily available up to 5000 K from the JANAF table (http://kinetics.nist.gov/janaf/)[25], whereas for Fe_2O_3 only that of the solid phase hematite is given. We, therefore, calculate the free energy of the reaction, ΔG_r^0 as follows. The ΔG_r^0 at the melting point of hematite at 1 bar and its melting temperature, T_m (i.e., 1895 K) [25] is expressed as

$$\Delta G_\mathrm{r}^0(T_\mathrm{m}) = \Delta G_\mathrm{f}^0(T_\mathrm{m,}\mathrm{FeO}_{1.5}, l) - \Delta G_\mathrm{f}^0(T_\mathrm{m,}\mathrm{FeO}, l)$$

with

$$\Delta G_\mathrm{f}^0(T_\mathrm{m,}\mathrm{FeO}_{1.5}, l) = \Delta G_\mathrm{f}^0(T_\mathrm{m,}\mathrm{FeO}_{1.5}, s)\ ,$$

where *l/s* represent liquid and solid phases, respectively. $\Delta G_\mathrm{f}^0(T_\mathrm{m,}\mathrm{Fe_2O_3}, s)$ and $\Delta G_\mathrm{f}^0(T_\mathrm{m,}\mathrm{FeO}, l)$ are given by the JANAF table. Note that the formation energy of the oxygen gas is by definition 0 J mol^{-1} and thus is neglected in the above expression. $\Delta G_\mathrm{r}^0(T)$ can be further related to its value at melting point by

$$\Delta G_\mathrm{r}^0(T) = \Delta G_\mathrm{r}^0(T_\mathrm{m}) - \int_{T_\mathrm{m}}^{T} \Delta S_\mathrm{r}^0(T)dt,$$

where ΔS_r^0 is the entropy of the reaction (1) and may be expressed as

$$\Delta S_\mathrm{r}^0 = S(\mathrm{FeO}_{1.5}, l) - S(\mathrm{FeO}, l) - 1/4S(\mathrm{O}_2, g).$$

Of the three right-hand terms, only the entropy of $FeO_{1.5}$ is not directly available from the JANAF table. The entropy of liquid $FeO_{1.5}$ at T_m, $S(T_\mathrm{m,}\ \mathrm{FeO}_{1.5}, l)$ can be calculated with the entropy of solid $S(T_\mathrm{m,}\ \mathrm{FeO}_{1.5}, s)$ and the entropy of melting of solid $FeO_{1.5}$ at 1 bar[14]. Similar to $\Delta G_r^0(T)$, the entropy of liquid $FeO_{1.5}$, $S(T, \mathrm{FeO}_{1.5}, l)$ can be related to that at melting temperature, $(T_\mathrm{m,}\ \mathrm{FeO}_{1.5}, l)$ by

$$S(T, \mathrm{FeO}_{1.5}, l) = S(T_\mathrm{m,}\ \mathrm{FeO}_{1.5}, l) + \int_{T_\mathrm{m}}^{T} \frac{C_P}{t} dt,$$

where C_P is the specific heat of liquid $FeO_{1.5}$, which was experimentally determined to be 120.45 ± 3.95 J mol^{-1} K^{-1} [26] and likely varies marginally at high temperatures (close to or beyond T_m)[27]. Therefore, we treat C_P to derive $S(T, \mathrm{FeO}_{1.5}, l)$, with which $\Delta G_\mathrm{r}^0(T)$ can be easily calculated. The result is plotted in Supplementary Fig. 5, in good agreement with previous models at lower temperatures of ~ 1000-2000 K [14,15]. At higher temperatures (>2000 K), extrapolations of previous models differ from our results and from each other. We stress that our model utilize the thermodynamic dataset of chemical species temperature as high as 5000 K and can thus be applied to 5000 K without extrapolation, while previous models are only directly applicable at <2100 K.

We then parameterize $\Delta G_\mathrm{r}^0(T)$ following the formulation proposed by ref.[28],

$$\Delta G_\mathrm{r}^0(T) = a + bT + cT\ln T + dT^2 + eT^{-1} + fT^{0.5},$$

where T is temperature in K, $\Delta G_\mathrm{r}^0(T)$ is in J mol^{-1}, and a = $-3.310\times10^5 \pm 172$, b= -190.379 ± 0.484, c = 14.785 ± 0.086, d = $-1.649\times10^{-3}\pm4.4\times10^{-5}$, e = $9.348\times10^6\pm1.2\times10^3$, f = $1.077\times10^4 \pm1.44$.

Supplementary Note 2

For the activities of $FeO_{1.5}$ and FeO in silicate melt, we consider a symmetric regular solution model:

$$\ln\frac{\gamma_{FeO_{1.5}}^{melt}}{\gamma_{FeO}^{melt}} = \sum_j^n X_j(W_{\mathrm{FeO_{1.5}-j}} - W_{\mathrm{FeO-j}})/RT + (X_{\mathrm{FeO}} - X_{\mathrm{FeO_{1.5}}})W_{\mathrm{FeO-FeO_{1.5}}}/RT\ ,$$

where W_{i-j} denotes the interaction Margules parameter of components i and j in the melt [14] and is treated as fitting parameters here. Previous experiments on the redox states of silicate melts cover a wide range of compositions, temperatures (1278-2300 K), and pressures (1 bar to 23 GPa) (Supplementary Table 2). Silicate melts studied generally resemble terrestrial magmas with major components of SiO_2, Al_2O_3, FeO, Fe_2O_3, MgO, CaO, K_2O, Na_2O, TiO_2, and P_2O_5. The available experimental data includes 225 data points at 1 bar[8,14,29-31] and 32 data points at higher pressures[4,5,16]. We do not include some 1-bar data for which the oxygen fugacities were not given in the source[8,32], although the best-fitting expressions were provided.

We first fit all available 1-bar data[8,14,29-31] and higher pressure dataset by refs.[5,16] (excluding ref. 4) to Eq. (3) in order to resolve the interaction parameters involved, denoted as Fit 1. The reduced chi-square is 0.063 and best-fitting parameters are tabulated in Supplementary Table 3. The comparisons between our predictions based on Fit 1 and experimental results are shown in Supplementary Fig. 6a. We also consider the fitting including a recently published high-pressure dataset[4] and results are shown in Supplementary Fig. 6b. This fitting (Fit 2) results in larger reduced chi-square, i.e., 2.774 than that of Fit 1 (0.064). This is mainly due to the relatively large difference between the prediction and the experimental results of ref. [4] at 10 GPa and pressures greater than 20 GPa (see Supplementary Note 5 for more discussion). Nevertheless, Fit 1 and Fit 2 yield similar values for the interaction parameters (Supplementary Table 3) and thus the redox profiles of the MO (Supplementary Fig. 7). In other words, Fit 1, based on the data up to 7 GPa can sufficiently predict the higher pressure experimental results by ref. [4] and inclusion of ref. 1 do not appear to make any appreciable differences (Supplementary Fig. 7). Note that Supplementary Fig. 6 is not intended to examine the high-pressure data, but it provides valuable comparison between the model prediction and experimental results at 1 bar. More detailed comparison between the model prediction and high-pressure experiments is considered in Supplementary Fig. 8.

In addition, three fitted parameters, $W_{\mathrm{FeO_{1.5}-NaO_{0.5}}} - W_{\mathrm{FeO-NaO_{0.5}}}$, $W_{\mathrm{FeO_{1.5}-PO_{2.5}}} - W_{\mathrm{FeO-PO_{2.5}}}$ and $W_{\mathrm{FeO_{1.5}-TiO_2}} - W_{\mathrm{FeO-TiO_2}}$ are poorly constrained in Fit 1 and Fit 2 with uncertainties much larger than the magnitude of the best-fitting values, suggesting that these components have little influence on the data fits. This is expected as contents of $NaO_{0.5}$, TiO_2, and $PO_{2.5}$ in previous experiments varies marginally. As such, we also consider excluding these three parameters and re-fit the experimental data without the dataset of by ref. [4] (denoted as Fit 3) and including ref.[4] (denoted as Fit 4). The goodness of the fitting is slightly better (Supplementary Fig. 6). Overall, the interaction parameters resolved using the four different fitting schemes are similar and thus generate similar redox profiles of MOs (Supplementary Fig. 7). In the main text, we show the redox state of the MO calculated using the interaction parameters resolved by Fit 3 since it has the smallest reduced chi-square (χ_{v}^2).

For low Fe^{3+}/Fe^{2+} samples, our model predictions (Fit 1, 2 ,3, 4 in Supplementary Fig. 6) deviate notably from some experimental results, mostly by Kress and Carmichael (1988)[31]. This

disagreement may be caused by the following. First, experimental results for ferric iron content of those samples by Kress and Carmichael (1988)[31] may be inaccurate due to technical difficulties at the time. In fact, Kress and Carmichael (1991)[8] remeasured the ferric iron content of some samples in Kress and Carmichael (1988) [31] and found the new results differ significantly from those reported by Kress and Carmichael (1988) [31]; Second, MnO component is lacking in our models. The MnO contents of those experimental samples exhibit a moderately positive correlation with the deviations of model predictions, indicating that MnO may be a key component that controls the Fe^{3+}/Fe^{2+} ratio when the Fe^{3+} is low. Future studies are warranted to explore this effect. For this study, due to the insufficient experiments with MnO bearing samples, we are not able to include the MnO component in our model. Nevertheless, MnO is a trace/minor component for Earth, Mars, and the Moon and thus not of interest here.

Supplementary Note 3

To further investigate the errors of our model predictions, we use fitted parameters from Fit3 as an example to calculate $Fe^{3+}/\Sigma Fe$ at the experimental conditions reported by previous high pressures studies[4,5,16].

The previous high-pressure experimental data are selected as follows. We adopt all the six high pressure data by ref. [16]. We also use all 19 experimental results by [5]. For the recent high pressure study by ref. [4], we take into account all the data shown in its Figure 1 and the corresponding ones with possible Ru contribution removed shown in their Figure S7. They are S6928, S6889, S6820, Z1794, Z1468, Z1621, Z1666, S6654, S6606, S6776, and S6973 (in total 11 samples). Samples S6879, S6811, S6510, and S6977 are excluded because they suffered from inhomogeneous Pt contamination of the oxygen buffer and are classified as unreliable results by ref. [4].

At low pressures (0-3 GPa), our predicted $Fe^{3+}/\Sigma Fe$ are around 0.1 lower than those of ref.[16] while in better agreement with those in ref.[5]. Zhang et al.[5] suggested that this discrepancy may result from the precision problems when sextets present in Mössbauer spectra, which is the case for all of data by O'Neill et al.[16]. Our prediction is in excellent agreement with experimental results at pressures from 3 to 6 GPa and the agreement deteriorates from 6 GPa to 10 GPa. It is noted that 6 to 10 GPa is where the quenched samples transition from glassy to crystalline and the ferric iron content of the system may be affected by this crystallization process. However, the corrections applied by Armstrong et al. [4] generally lower the ferric iron content and thus would make the agreement worse. From 15 to 20 GPa, our calculated Fe^{3+}/Fe^{2+} exhibit good agreement with the experimental results. At the highest pressure reached (23 GPa) by Armstrong et al. [4], two runs with the similar bulk compositions and oxygen fugacity show almost 100% ferric iron content. The correction that removes the possible Ru contributions lowers these values by ~10%, which are still ~10% higher than the predicted ones. Nevertheless, our model prediction is consistent with the experimental observation within the uncertainties and the variation trend with pressure (decrease followed by increase) is consistent with experiments. The same goes for the variation of Fe^{3+}/Fe^{2+} with temperature. Overall, our model shows a shallower variation trend with pressures compared with that of Armstrong et al. [4]. This is caused by our more gradual variation of ΔV especially at pressures higher than 10 GPa (Supplementary Fig. 1).

We note the comparison between the model prediction and experiments may suffer from the paucity of high-pressure studies, especially at pressures around 10 GPa and higher than 20 GPa. More experiments at these pressures may help better evaluate the model and elucidate the high-pressure redox buffer reaction.

Supplementary Tables

Supplementary Table 1. Fitted equation of state parameters for silicate melts containing 12.5 mol% ($Mg_{14}Fe_2Si_{16}O_{48}$ and $Mg_{14}Fe_2Si_{16}O_{49}$) and 25 mol% ($Mg_{12}Fe_4Si_{16}O_{48}$ and $Mg_{12}Fe_4Si_{16}O_{50}$) iron (Eq. 2). The reference temperature is 3000 K. Uncertainties are given in parentheses.

	V_0 (A^3)	K_0 (GPa)	K'	K'' (GPa^{-1})	a	b	c	χ^2_v
$Mg_{14}Fe_2Si_{16}O_{48}$	1180.10 (0.33)	26.76 (0.05)	2.80 (0.01)	0.01 (0.00)	35.70 (0.04)	71.10 (0.06)	36.60 (0.04)	0.0002
$Mg_{14}Fe_2Si_{16}O_{49}$	1204.69 (0.55)	23.18 (0.07)	3.22 (0.01)	0.01 (0.00)	34.53 (0.03)	68.64 (0.07)	35.27 (0.04)	0.0004
$Mg_{12}Fe_4Si_{16}O_{48}$	1192.01 (0.43)	23.95 (0.07)	3.32 (0.01)	–0.01 (0.00)	31.35 (0.11)	62.49 (0.27)	32.47 (0.17)	0.0003
$Mg_{12}Fe_4Si_{16}O_{50}$	1256.73 (0.85)	16.13 (0.10)	4.58 (0.02)	–0.18 (0.00)	30.38 (0.10)	59.11 (0.26)	29.65 (0.16)	0.0004

Supplementary Table 2. Summary of previous experiments on the redox states of silicate melts.

P(GPa)	*T* (K)	log f_{O_2}	# data points	References
10^{-4}	1468-1836	–8.1 - –5.07	62[a]	Thornber et al. (1980)
10^{-4}	1474-1603	–8.46 - –6.77	57	Sack et al. (1980)
10^{-4}	1633 - 1734	–10.23 - –0.58	48[b]	Kress and Carmichael (1988)
10^{-4}	1522 - 1908	–6.29 - –0.68	13[c]	Kress and Carmichael (1991)
10^{-4}	1278 - 1830	–10.4 - –0.67	29	Moore et al. (1995)
10^{-4}	1673	–6.91 - –0.01	15	Jayasuriya et al. (2004)
0.4 - 3	1673	–0.60 - 0.28	6	O'Neill et al. (2006)
10^{-4} - 7	1673 - 2023	–0.72 - 2.62	19	Zhang et al. (2017)
4 - 23	1673-2300	0.53 -7.02	11[d]	Armstrong et al. (2019)

* a, the Al_2O_3 content of run#401 is incorrectly reported in their table and should be 18.68 based on the descriptions in their main text.
* b, run#B27-B210 were re-measured in Kress and Carmichael.[8] and not included this study.
* c, the f_{O_2} of the run# Q01 - Q66 were not given in the source and thus not included here.
* d, we take into account all the data shown in Figure 1 of Armstrong et al. [4]. They are S6928, S6889, S6820, Z1794, Z1468, Z1621, Z1666, S6654, S6606, S6776, and S6973.
* Kilinc et al.[32] presented 46 1-bar results but failed to provide the raw f_{O_2} data and thus are ignored here.

Supplementary Table 3. Best-fitting interaction parameters in J/mol with 1σ standard deviations given in the parentheses. Fits 1 and 3, and Fits 2 and 4, denote fitting without and with the dataset by Armstrong et al. [4], respectively. $W_{FeO_{1.5}-NaO_{0.5}} - W_{FeO-NaO_{0.5}}$, $W_{FeO_{1.5}-PO_{2.5}} - W_{FeO-PO_{2.5}}$, and $W_{FeO_{1.5}-TiO_2} - W_{FeO-TiO_2}$ are set to 0 in Fit 3 and Fit 4.

	$W_{FeO1.5\text{-}j}$ - $W_{FeO\text{-}j}$								W_{FeO} - $W_{FeO1.5}$	data#	χ_v^2
j	MgO	SiO_2	$AlO_{1.5}$	CaO	$NaO_{0.5}$	$KO_{0.5}$	$PO_{2.5}$	TiO_2			
Fit 1	75011<br>(10200)	2029<br>(2738)	30836<br>(7026)	–65959<br>(4385)	0<br>(9279)	–49254<br>(20993)	1<br>(30047)	–9843<br>(15890)	–11552<br>(4867)	249	0.064
Fit 2	37352<br>(58515)	0<br>(14346)	42858<br>(43368)	–42218<br>(31915)	–21803<br>(119161)	–34275<br>(70368)	119741<br>(207879)	–105079<br>(106717)	–2<br>(30388)	260	2.774
Fit 3	68629<br>(4332)	4601<br>(1507)	40923<br>(3874)	–58109<br>(3967)	0<br>-	–59584<br>(9099)	0<br>-	0<br>-	–14210<br>(4876)	249	0.062
Fit 4	35409<br>(10333)	4101<br>(20001)	45924<br>(41900)	–50920<br>(31515)	0<br>-	–62943<br>(40852)	0<br>-	0<br>-	–8735<br>(24836)	260	2.967

Supplementary Table 4. Composition of terrestrial MOs used in our study, with all components are in wt%.

	SiO_2	Al_2O_3	FeO_T	MgO	CaO	K_2O	Na_2O	TiO_2	P_2O_5	Total	References
Earth	45	4.45	8.05	37.8	3.55	0.029	0.36	0.2	0.021	99.5	McDonough and Sun, 1995
Moon*	45.1	5.3	9.375	35.6	3.82	0.01	0.17	0.23	0.01	99.6	Elardo *et al.*, 2011
Mars	43.68	3.13	18.71	31.5	2.49	0	0.5	0	0	100.0	Bertka and Fei, 1997

* The lunar MO is the average of Taylor Whole Moon (TWM) model and Lunar Primitive Upper Mantle (LPUM)[33].

Supplementary References

1 deKoker, N. & Stixrude, L. Self-consistent thermodynamic description of silicate liquids, with application to shock melting of MgO periclase and $MgSiO_3$ perovskite. *Geophys J Int* **178**, 162-179, doi:10.1111/j.1365-246X.2009.04142.x (2009).

2 Liebske, C. & Frost, D. J. Melting phase relations in the MgO-$MgSiO_3$ system between 16 and 26 GPa: Implications for melting in Earth's deep interior. *Earth and Planetary Science Letters* **345**, 159-170, doi:10.1016/j.epsl.2012.06.038 (2012).

3 Stixrude, L., de Koker, N., Sun, N., Mookherjee, M. & Karki, B. B. Thermodynamics of silicate liquids in the deep Earth. *Earth and Planetary Science Letters* **278**, 226-232, doi:10.1016/j.epsl.2008.12.006 (2009).

4 Armstrong, K., Frost, D. J., McCammon, C. A., Rubie, D. C. & Boffa Ballaran, T. Deep magma ocean formation set the oxidation state of Earth’s mantle. *Science* **365**, 903-906, doi:10.1126/science.aax8376 (2019).

5 Zhang, H. L., Hirschmann, M. M., Cottrell, E. & Withers, A. C. Effect of pressure on $Fe^{3+}/\Sigma Fe$ ratio in a mafic magma and consequences for magma ocean redox gradients. *Geochimica et Cosmochimica Acta* **204**, 83-103, doi:10.1016/j.gca.2017.01.023 (2017).

6 Lange, R. A. & Carmichael, I. S. E. Densities of Na_2O-K_2O-CaO-MgO-FeO-Fe_2O_3-Al_2O_3-TiO_2-SiO_2 liquids: New measurements and derived partial molar properties. *Geochimica et Cosmochimica Acta* **51**, 2931-2946, doi:10.1016/0016-7037(87)90368-1 (1987).

7 Lange, R. L. & Carmichael, I. S. E. Thermodynamic properties of silicate liquids with emphasis on density, thermal expansion and compressibility. *Reviews in Mineralogy and Geochemistry* **24**, 25-64 (1990).

8 Kress, V. C. & Carmichael, I. S. E. The compressibility of silicate liquids containing Fe_2O_3 and the effect of composition, temperature, oxygen fugacity and pressure on their redox states. *Contributions to Mineralogy and Petrology* **108**, 82-92, doi:10.1007/BF00307328 (1991).

9 Drewitt, J. W. E., Sanloup, C., Bytchkov, A., Brassamin, S. & Hennet, L. Structure of $(Fe_xCa_{1-x}O)_y(SiO2)_{1-y}$ liquids and glasses from high-energy x-ray diffraction: Implications for the structure of natural basaltic magmas. *Phys Rev B* **87**, 224201, doi:10.1103/PhysRevB.87.224201 (2013).

10 Wilke, M., Partzsch, G. M., Bernhardt, R. & Lattard, D. Determination of the iron oxidation state in basaltic glasses using XANES at the K-edge. *Chem Geol* **220**, 143-161, doi:10.1016/j.chemgeo.2005.03.004 (2005).

11 Alderman, O. L. G. *et al.* Iron K-edge X-ray absorption near-edge structure spectroscopy of aerodynamically levitated silicate melts and glasses. *Chem Geol* **453**, 169-185, doi:10.1016/j.chemgeo.2017.01.020 (2017).

12 Sanloup, C. Density of magmas at depth. *Chem Geol* **429**, 51-59, doi:10.1016/j.chemgeo.2016.03.002 (2016).

13 Sanloup, C. *et al.* Structure and density of molten fayalite at high pressure. *Geochimica et Cosmochimica Acta* **118**, 118-128, doi:10.1016/j.gca.2013.05.012 (2013).

14 Jayasuriya, K. D., Campbell, S. J., Berry, A. J. & O’Neill, H. S. C. A Mössbauer study of the oxidation state of Fe in silicate melts. *American Mineralogist* **89**, 1597-1609, doi:10.2138/am-2004-11-1203 (2004).

15 Gaillard, F., Pichavant, M. & Scaillet, B. Experimental determination of activities of FeO and Fe_2O_3 components in hydrous silicic melts under oxidizing conditions. *Geochimica et Cosmochimica Acta* **67**, 4389-4409, doi:10.1016/S0016-7037(03)00376-4 (2003).

16 O'Neill, H. S. C. *et al.* An experimental determination of the effect of pressure on the $Fe^{3+}/\Sigma Fe$ ratio of an anhydrous silicate melt to 3.0 GPa. *American Mineralogist* **91**, 404-412, doi:10.2138/am.2005.1929 (2006).

17 Fischer, R. A. *et al.* High pressure metal–silicate partitioning of Ni, Co, V, Cr, Si, and O. *Geochimica et Cosmochimica Acta* **167**, 177-194, doi:10.1016/j.gca.2015.06.026 (2015).

18 Yang, S. *et al.* Siderophile and chalcophile element abundances in shergottites: Implications for Martian core formation. **50**, 691-714, doi:10.1111/maps.12384 (2015).

19 Garcia, R. F., Gagnepain-Beyneix, J., Chevrot, S. & Lognonne, P. Very Preliminary Reference Moon Model (vol 188, pg 96, 2011). *Physics of the Earth and Planetary Interiors* **202**, 89-91 (2012).

20 Li, J. & Agee, C. B. Geochemistry of mantle–core differentiation at high pressure. *Nature* **381**, 686-689, doi:10.1038/381686a0 (1996).

21 Cartier, C. & Wood, B. J. The Role of Reducing Conditions in Building Mercury. *Elements* **15**, 39-45, doi:10.2138/gselements.15.1.39 (2019).

22 Frost, D. J. & McCammon, C. A. The redox state of Earth's mantle. *Annu Rev Earth Pl Sc* **36**, 389-420 (2008).

23 Herd, C. D. K. The oxygen fugacity of olivine-phyric martian basalts and the components within the mantle and crust of Mars. *Meteorit Planet Sci* **38**, 1793-1805, doi:10.1111/j.1945-5100.2003.tb00015.x (2003).

24 Dyar, M. D. Ferric iron in SNC meteorites as determined by Mössbauer spectroscopy: Implications for martian landers and martian oxygen fugacity. **38**, 1733-1752, doi:10.1111/j.1945-5100.2003.tb00012.x (2003).

25 Chase, M. W., National Institute of, S. & Technology. *NIST-JANAF thermochemical tables*. (American Chemical Society ; American Institute of Physics for the National Institute of Standards and Technology, 1998).

26 Lange, R. A. & Navrotsky, A. Heat capacities of Fe_2O_3-bearing silicate liquids. *Contributions to Mineralogy and Petrology* **110**, 311-320, doi:10.1007/BF00310746 (1992).

27 Granato, A. V. The specific heat of simple liquids. *J Non-Cryst Solids* **307-310**, 376-386, doi:10.1016/S0022-3093(02)01498-9 (2002).

28 Komabayashi, T. Thermodynamics of melting relations in the system Fe-FeO at high pressure: Implications for oxygen in the Earth's core. *Journal of Geophysical Research: Solid Earth* **119**, 4164-4177, doi:10.1002/2014JB010980 (2014).

29 Thornber, C. R., Roeder, P. L. & Foster, J. R. The effect of composition on the ferric-ferrous ratio in basaltic liquids at atmospheric pressure. *Geochimica et Cosmochimica Acta* **44**, 525-532, doi:10.1016/0016-7037(80)90048-4 (1980).

30 Sack, R. O., Carmichael, I. S. E., Rivers, M. & Ghiorso, M. S. Ferric-ferrous equilibria in natural silicate liquids at 1 bar. *Contributions to Mineralogy and Petrology* **75**, 369-376, doi:10.1007/BF00374720 (1981).

31 Kress, V. C. & Carmichael, I. S. E. Stoichiometry of the Iron Oxidation Reaction in Silicate Melts. *American Mineralogist* **73**, 1267-1274 (1988).

32 Kilinc, A., Carmichael, I. S. E., Rivers, M. L. & Sack, R. O. The ferric-ferrous ratio of natural silicate liquids equilibrated in air. *Contributions to Mineralogy and Petrology* **83**, 136-140, doi:10.1007/BF00373086 (1983).

33 Elardo, S. M., Draper, D. S. & Shearer, C. K. Lunar Magma Ocean crystallization revisited: Bulk composition, early cumulate mineralogy, and the source regions of the highlands Mg-suite. *Geochimica Et Cosmochimica Acta* **75**, 3024-3045, doi:Doi 10.1016/J.Gca.2011.02.033 (2011).
